# Characteristics of Hemorrhagic Stroke following Spine and Joint Surgeries

**DOI:** 10.1155/2017/5390839

**Published:** 2017-01-10

**Authors:** Fei Yang, Jianning Zhao, Haidong Xu

**Affiliations:** Department of Orthopedics, School of Medicine, Jinling Hospital, Nanjing University, Nanjing, Jiangsu 210002, China

## Abstract

Hemorrhagic stroke can occur after spine and joint surgeries such as laminectomy, lumbar spinal fusion, tumor resection, and total joint arthroplasty. Although this kind of stroke rarely happens, it may cause severe consequences and high mortality rates. Typical clinical symptoms of hemorrhagic stroke after spine and joint surgeries include headache, vomiting, consciousness disturbance, and mental disorders. It can happen several hours after surgeries. Most bleeding sites are located in cerebellar hemisphere and temporal lobe. A cerebrospinal fluid (CSF) leakage caused by surgeries may be the key to intracranial hemorrhages happening. Early diagnosis and treatments are very important for patients to prevent the further progression of intracranial hemorrhages. Several patients need a hematoma evacuation and their prognosis is not optimistic.

## 1. Introduction

The definition of stroke is “rapidly developing clinical signs of focal disturbance of cerebral function, with symptoms lasting 24 hours or longer or leading to death, with no apparent cause other than of vascular origin” by WHO (The World Health Organization) [1]. Stroke can happen to any person at any time. It happens when poor blood flow to an area of brain, and brain cells begin to die because of lack of oxygen. Abilities such as memory and muscle control controlled by that area of the brain are damaged. According to the National Stroke Association, nearly 800,000 people experience a new or recurrent stroke each year. Stroke is the fifth leading cause of death and the leading cause of adult disability in USA. According to the pathology, stroke can be divided into two main types: ischemic and hemorrhagic. Hemorrhagic strokes are relatively uncommon compared with ischemic strokes. Actually hemorrhagic strokes account for only 15% of all strokes; however 40% of all stroke deaths are attributable to hemorrhagic strokes [2]. The common causes of hemorrhagic strokes include arteriolar hypertensive diseases, burst aneurysm, arteriovenous malformation (AVM), bleeding disorders, head injury, and blood thinners. In addition to this, some clinical scientists find that hemorrhagic strokes sometimes occur after spine and joint surgeries. Although postoperative stroke rarely happens, it may cause severe consequences and high mortality rates. Chadduck first reported a case of hemorrhagic stroke after cervical laminectomy [3]. Since then, there are several similar cases reported. And most patients in these cases have headache, neurological disorders, and altered level of consciousness after spine surgery. The bleeding site is usually located in cerebellum after spine surgeries. Finally some patients in these cases become disabled and even dead. Hemorrhagic strokes after joint surgeries are also the cause of disability and death. Nevertheless, we know very little about these kinds of hemorrhagic stroke now, and it is necessary to study it in depth. This review summarizes the clinical status, risk factors, pathologic mechanisms, and treatment options of perioperative hemorrhagic stroke after spine and joint surgeries.

## 2. Methods

A computer-based retrieval was performed to search articles which describe hemorrhagic stroke after spine and joint surgeries published between September 1, 1980, and September 1, 2016, in PubMed database with the key words of “stroke, spine, joint” in English. And 141 articles were found in this way. The inclusion criteria includes the following. (1) Studies should cover the clinical status, risk factors, and treatment options of hemorrhagic stroke after spine and joint surgeries. (2) Study assumptions and research methods are similar. (3) The patient's diagnosis is clear. The exclusion criteria includes repeated reports, incomplete data, and study defect. After filtering these articles by the inclusion and exclusion criteria, finally, 25 articles are included into this review.

## 3. Results

### 3.1. Clinical Status

Postoperative intracranial hemorrhages may happen after spinal surgery in different places in the brain, such as the epidural or subdural space and the supratentorial or cerebellar parenchyma. Although it is a rare complication, it can be related to permanent serious disability [4]. Chadduck reported the first case of remote cerebellar hemorrhage (RCH) of a patient who had undergone a cervical laminectomy in the sitting position. The clinical manifestations of this patient included headache, cerebellar neurological disorders, and altered level of consciousness [3]. After that Mikawa reported the second case: a patient became comatose almost 16 hours after cervical durotomy and revision C1-C2 fusion, as the cerebellar hemorrhage occurred within the first 10 hours after the surgery. From the first case reported to the present, there are 44 published cases of remote hemorrhage reported after spinal surgery. Among these, there are 11 cases undergoing cervical laminectomy, 19 cases undergoing lumbar laminectomy, 10 cases undergoing lumbar spinal fusion, 3 cases undergoing tumor resection, and 1 case undergoing Harington rod placement [5–10]. In these cases, most patients had clinical manifestations of headache, vomiting, consciousness disturbance, and mental disorders, and the CT scan of these patients showed subarachnoid hemorrhage or hemorrhage of the brain parenchyma. Most patients can recover after active treatment and rehabilitation exercise among these cases; however there are also some patients becoming disabled and even dead (Table 1). In order to verify whether spine surgery is associated with stroke, Jau-Ching Wu conducted a cohort study in Taiwan. In this study, a Taiwan-wide cohort of 1 million people from 2000 to 2005 was divided into the lumbar spinal fusion group and they were followed up for 3 years for stroke. The result shows that patients undergoing lumbar spinal fusion do not have a higher incidence rate of stroke. And the author admits that the result of this study is not convincing enough because of the database limits. So it is still unclear whether spine surgery and stroke are related. Hemorrhagic stroke is also a disastrous complication after joint surgeries such as total joint arthroplasty (TJA). Rasouli et al. made a database review which covered a total of 1,762,496 patients after TJA from 2002 to 2011. After doing a population-based trend analysis, they found that the incidence rate of all perioperative stroke after TJA was nearly 0.14%. And among these perioperative strokes, 20.55% of cases were hemorrhagic stroke. The in-hospital mortality rate was much higher for TJA patients with stroke than patients without stroke (9% versus 0.15%) [11]. Although the epidemiological studies showed a low frequency of perioperative stroke, it is still the leading cause of disability and death for patients after joint surgeries [12].

### 3.2. Risk Factors

The risk factors of a common stroke include dyslipidemia, hypertension, diabetes mellitus, smoking, and obesity. These risk factors also exist in patients after spine and joint surgeries [13]. For patients after spine surgeries, hypertension and coagulopathy are considered as main risks of hemorrhagic stroke [14]. And low intracranial pressure (ICP) may contribute to intracranial hemorrhages especially subdural hemorrhages [15]. Other important risks include ages of patients and experience of surgeons, and these risks are doubled if a patient once had an experience of a disc surgery [16]. For patients after joint surgeries, the main risks include diabetes mellitus, cardiac diseases, renal diseases, and pulmonary circulation disorders. A history of stroke is not risk factor for contributing to stroke after joint surgeries [11]. There is also a higher incidence of first-ever stroke for patients who are over 65 years old after hip replacement surgeries, and the incidence rate of ischemic stroke is nearly five times than that of hemorrhagic stroke [17]. It is worth mentioning that adiposity-associated risks of women are much greater for ischemic stroke than for hemorrhagic stroke [18].

### 3.3. Pathologic Mechanisms

The exact pathophysiology of intracranial hemorrhages after spine surgeries is still controversial. However almost all of the theories are associated with cerebrospinal fluid (CSF) leakage which leads to intracranial hypotension [19]. And dura mater tears which can cause CSF leakage are the most common complications in a spine surgery [20]. One of these theories for hemorrhagic stroke after CSF leakage suggests that a downward cerebellar displacement may happen following with the CSF leakage and intracranial hypotension. And the downward cerebellar displacement can lead to the stretching and tearing of cerebral venous system which finally causes hemorrhagic stroke [21]. Another theory believes that the pressure in brain vessels increases after CSF leakage and ruptures the vessels [22]. There are few studies on the pathophysiology of intracranial hemorrhages after joint surgeries because of its low occurrence. So the pathophysiology of intracranial hemorrhages after joint surgeries is not very clear now. With more research conducted and more cases reported, the pathophysiology will be revealed finally.

### 3.4. Prevention Strategies

According to the currently known risk factors, some prevention strategies can be implemented to prevent patients from hemorrhagic strokes after spine and joint surgeries. Firstly, blood pressure control must be performed throughout the perioperative period especially for those patients with hypertension, because hypertension not only increases the risk of surgery but also increases the incidence of postoperative complications. Secondly, the operator should try to avoid tearing the dura mater during the spine surgery. Once a CSF leakage happens, intracranial pressure monitor will be useful to evaluate the risk of hemorrhagic stroke. And some measures such as dural repair should be done before hemorrhagic stroke happens. For patients after tumor resection, wound suction drainage is a double-edged sword. On the one hand it can reduce intracranial edema, but on the other hand it causes CSF leakage and increases the risk of intracranial hypotension. So the correct choice must be made based on the patient's comprehensive circumstance. Thirdly, early brain CT examinations are necessary for patients with vascular disease or coagulopathy. Finally, the treatment of underlying diseases may also be beneficial to prevent hemorrhagic stroke.

### 3.5. Treatments

For patients with hemorrhagic stroke after spine surgeries, it is the most important that the CSF leakage and intracranial hypotension must be controlled. So closed wound suction drainage is recommended for spinal surgery especially tumor resection, and the time to stop drainage is determined by the complaints of patients including headache and emesis [8]. In other words, early diagnosis is also very important for the treatment of intracranial hemorrhage after spinal surgery. Brain CT examinations and typical clinical symptoms may contribute to early diagnosis. If the loss of CSF is caused by a dural tear, dural repair and preventing CSF leakage will be useful to prevent intracranial hemorrhages after spine surgeries. Other treatments including symptomatic treatment and supportive care in a common hemorrhagic stroke such as bed rest, clinical intensive observation, fluid therapy, and radiological close monitoring are also necessary for patients with hemorrhagic stroke after spine and joint surgeries [23]. Rational application of mannitol can reduce intracranial hypertension after hemorrhagic stroke happens. Antihypertensive drugs is beneficial to patients with hypertension, whether hemorrhagic stroke happens or not. Other drugs such as hypoglycemic agent for underlying diseases are also necessary. And new drugs such as Fingolimod (FTY720) may be useful to treat hemorrhagic stroke after spine and joint surgeries [24], because the latest laboratory findings and clinical trials strongly support its effectiveness on any kind of hemorrhagic stroke [25].

## 4. Conclusions

Hemorrhage stroke after spine and joint surgeries is relatively rare, but it may cause serious consequences such as morbidity and mortality. In addition to dyslipidemia, hypertension, diabetes mellitus, smoking, and obesity, the risks of patients after surgeries include coagulopathy and low intracranial pressure. Most patients with hemorrhage stroke after surgeries have clinical manifestations of headache, vomiting, consciousness disturbance, and mental disorders. The bleeding sites are mostly located in cerebellar hemisphere and temporal lobe. Most cases happen several hours after surgeries. And brain CT examinations and typical clinical symptoms may contribute to early diagnosis. A CSF leakage may be the key to intracranial hemorrhages happening. So repairing dural tear or closing wound suction drainage after spine surgeries can be helpful to prevent hemorrhage stroke. Blood pressure control is very important for patients with hypertension. And other treatments such as bed rest, clinical intensive observation, fluid therapy, and radiological close monitoring are also necessary. Most patients can completely recover with no neurologic defect after the conservative treatment. However several patients need a hematoma evacuation. For these patients, their prognosis is not optimistic.

## Figures and Tables

**Table 1:** Clinical status of hemorrhagic stroke after different types of spine surgeries.

Surgery types	Clinical manifestations	CT appearance	Brain parenchyma hemorrhage location	Treatments	Results	Total case
Cervical laminectomy	10 cases have a serious headache, 1 case has aphasia, and 2 cases have limb motor dysfunction	3 cases show subarachnoid hemorrhage, and 8 cases show brain parenchyma hemorrhage	4 cases locate unilateral cerebellar hemisphere, 3 cases locate unilateral temporal lobe, and 1 case locates bilateral cerebellar hemisphere	8 cases under conservative treatments, 2 cases under dural tear repairing, and 1 case under decompressive craniectomy	9 cases completely recover with no neurologic defect, 1 case recovers with lower limbs spasticity, and 1 case died	11
Lumbar laminectomy	17 cases have headache and nausea, 6 cases have consciousness disturbance, 3 cases have limb motor dysfunction, and 1 has gait ataxia	4 cases show subarachnoid hemorrhage, 12 cases show brain parenchyma hemorrhage, and 1 case shows both of them	8 cases locate unilateral cerebellar hemisphere, 2 cases locate cerebellar vermis, 1 case locates right temporal lobe, and 1 case locates parietooccipital lobes	11 cases under conservative treatments, 4 cases under dural tear repairing, 2 cases under hemorrhage evacuation, and 2 cases under decompressive craniectomy	15 cases completely recover with no neurologic defect, 2 cases died, 1 case has left foot drop and diplopia, and 1 case has cognitive deficit	19
Lumbar spinal fusion	All cases have headache and nausea, 2 cases have dysarthria, 1 case has a speech deficit, and 1 case has consciousness disturbance	1 case shows subarachnoid hemorrhage, and 7 cases show brain parenchyma hemorrhage, and 2 cases show both of them	6 cases locate unilateral cerebellar hemisphere, 1 case locates bilateral cerebellar hemisphere, and 2 cases locate unilateral occipital lobe	7 cases under conservative treatments, 2 cases under dural tear repairing, and 1 case under hematoma evacuation	8 cases completely recover with no neurologic defect, 1 case has speech deficit, and 1 case died	10
Tumor resection	All cases have headache, and 1 has dizziness and vomiting	2 cases show cerebellar hemorrhage, and 1 case shows cerebral hemisphere hemorrhage	2 cases locate unilateral cerebellar hemisphere, and 1 case locates left temporooccipital cortex	All cases under conservative treatments	2 cases completely recover, and 1 case has a slight ataxia	3
Harington rod placement	Headache and vomiting	Subarachnoid hemorrhage and cerebellum hemorrhage	Right cerebellar hemispheres, right ventricle, and subarachnoid spaces	Suboccipital craniotomy	Completely recovered	1
